# Microplastics and Nanoplastics in the Human Intestine: A Review of Health Implications and Possible Dietary Interventions

**DOI:** 10.1016/j.advnut.2026.100624

**Published:** 2026-03-26

**Authors:** Alberto Dávalos, Victoria Martín-Santiago, María-Carmen López de las Hazas

**Affiliations:** 1 Laboratory of Epigenetics of Lipid Metabolism, Instituto Madrileño de Estudios Avanzados-Nutrición, CEI UAM+CSIC, Madrid, Spain; 2 Consorcio CIBER de la Fisiopatología de la Obesidad y Nutrición, Instituto de Salud Carlos III, Madrid, Spain; 3 Departament of Metabolism and Nutrition, Institute of Food Science, Technology and Nutrition, Spanish Research Council (ICTAN-CSIC), Madrid, Spain

**Keywords:** microplastics, nanoplastics, food safety, gastrointestinal tract, gut microbiota, human exposure, health risk

## Abstract

Microplastics and nanoplastics (MNPs) have emerged as pervasive dietary contaminants, yet their implications for human health remain poorly defined. This review synthesized current evidence on MNP interactions with the gastrointestinal system, emphasizing barrier disruption, biotransformation, particle uptake, and gut microbiota dysbiosis. Experimental data indicate that particle size, polymer type, and surface properties critically influence intestinal uptake and toxicity, with smaller particles showing greater systemic translocation. Although human evidence is limited and methodologies lack standardization, preliminary associations link MNP exposure with inflammatory bowel disease and metabolic disturbances. Beyond risks, we highlight the role of dietary patterns in modulating exposure and effects: minimizing processed, packaged foods and enhancing fiber, antioxidants, and probiotics may reduce intestinal burden and mitigate oxidative and inflammatory responses. Bridging mechanistic insights with dietary strategies, this review underscores the urgent need for standardized detection methods, human cohort studies, and sustainable food-system interventions.

**Statement of significance**

This review uniquely integrated mechanistic toxicology of dietary microplastics and nanoplastics with actionable nutritional strategies, providing the first concise synthesis that links intestinal biology and particle toxicodynamics to specific dietary interventions (e.g., fiber, antioxidants, and probiotics) and food-system measures aimed at reducing exposure and harm.

## Introduction

Plastics are integral to virtually every industry and aspect of daily life. Plastic pollution continues to increase. In 2020, ∼ 367 million metric tons of plastic waste were generated globally [1], and by ∼ 2050 ≤12 billion metric tons may enter landfills or the natural environment [2]. Plastic residues are formed of different polymers [i.e., polyethylene (PE), polypropylene (PP), polystyrene (PS), polyvinylchloride (PVC), and polyethylene terephthalate (PET)]. Physical, mechanical, chemical, and biological factors cause them to age and wear, generating fragments that can take various forms (i.e., fibers, films, fragments, particles, and foams) and sizes [1]. Microplastics (MPs) are particles <5 mm in diameter, and nanoplastics (NPs) those <1 μm; the threshold for NPs varies in the literature, in some research it is <100 nm. This lack of terminological consensus can make results comparison challenging. In this study, we refer to MPs as <5 mm, NPs <1 μm, and, for convenience, occasionally group them as MNPs (microplastics and nanoplastics).

As MNPs age and wear, the available plastic surface area increases, facilitating both particle translocation and entrance into biological matrices [3], and helping them adsorb other pollutants, specifically more hydrophobic ones. Other factors such as surface charge, functional groups, pathogen accumulation, exposure time, particle concentration, particle shape, and polymer type can also influence their potential toxicity [4,5].

Although human exposure to MNPs occurs through multiple pathways, including inhalation, dermal contact, and dietary intake, this review specifically focuses on ingestion as a predominant and continuous route of exposure for a significant fraction of MP and NPs [6]. Dietary sources, encompassing both contaminated foodstuffs/beverages and leaching from food contact materials, constitute a major input of MNPs directly into the gastrointestinal (GI) tract. Consequently, the intestinal epithelium represents a critical primary interface for a substantial portion of the human MNP burden. Therefore, the primary aim of this review is to synthesize current evidence on the interactions of diet-derived MNPs with the human GI system. We will focus specifically on their intestinal fate—including barrier disruption, cellular uptake mechanisms, and biotransformation—and their local and systemic health implications, particularly through modulation of gut microbiota. Furthermore, we evaluate evidence-based dietary and nutritional strategies that may mitigate exposure or counteract adverse effects. By delimiting our scope to the dietary exposure route and intestinal toxicodynamics, we aim to provide a focused analysis that complements broader systematic toxicology reviews and directly addresses the public health questions surrounding foodborne MNP contamination.

## Methods

This integrative review was conducted to synthesize current evidence on the interactions of dietary MNPs with the human GI tract, associated health implications, and potential dietary interventions. The methodology was designed to ensure transparency, reproducibility, and comprehensive coverage of the relevant literature.

### Information sources and search strategy

A systematic literature search was performed across 2 major electronic databases: PubMed and Scopus. The search timeframe spanned from database inception until October 2025 to capture the most recent findings in this rapidly evolving field. The search strategy combined controlled vocabulary (Medical Subject Headings (MeSH) terms, where applicable) and free-text keywords related to the core concepts of MPs, NPs, the GI tract, human health effects, and dietary factors. The following search string, adapted for each database, represents the core strategy: *(microplastic* OR nanoplastic∗ OR “micro plastic” OR “nano plastic” OR “plastic particle”*) AND (intestine* OR gastrointestinal OR “GI tract” OR gut OR colon OR fecal OR stool) AND (health OR tox∗ OR effect∗ OR impact∗ OR inflamm∗ OR disease*) AND (diet* OR food∗ OR nutrient∗ OR intake OR exposure OR consum∗ OR intervention OR mitigation)∗.

Additional manual searches of reference lists from eligible articles and recent key reviews were conducted to identify further relevant studies.

### Eligibility criteria

Studies were selected based on the following predefined criteria:

Inclusion criteria:

1) Original research articles, reviews, and meta-analyses published in English.
2) Studies investigating exposure to, or effects of, microplastics and/or nanoplastics (size defined as <5 mm).
3) Primary focus on the mammalian (including human) GI tract, intestinal barrier, gut microbiota, or related systemic metabolic/inflammatory effects.
4) Studies examining dietary sources of MNPs, dietary exposure assessment, or nutritional/dietary intervention strategies.
5) In vitro, in vivo (animal), and human observational/interventional studies.

Exclusion criteria:

1) Studies focusing exclusively on environmental fate, ecotoxicology in nonmammalian species, or nondietary exposure routes (e.g., inhalation, dermal) without relevance to intestinal uptake.
2) Conference abstracts, editorials, opinion pieces, or studies without primary data.
3) Studies where the polymer particles were not clearly identified as synthetic plastics.

### Study selection process

The study selection followed a structured, multistep process:

1) Identification: all retrieved records from database searches were combined, and duplicates were removed using reference management software.
2) Screening: titles and abstracts of all unique records were screened independently by 2 authors (AD and VM-S) against the eligibility criteria.
3) Full-text assessment: potentially relevant full-text articles were retrieved and assessed in detail for final inclusion. Discrepancies at any stage were resolved through discussion or by consultation with a third author (M-CLdlH).
4) Data extraction: data from included studies were extracted into a standardized template, capturing: author(s), publication year, study design (in vitro, animal, human), MNP characteristics (type, size, shape), exposure model/dose, key findings related to intestinal health, and proposed or tested dietary factors.

### Synthesis and quality consideration

Given the heterogeneity in study designs, exposure models, and outcomes, a formal meta-analysis or systematic review was not feasible. Instead, findings were synthesized thematically into narrative sections covering: *1*) MNP interaction with the intestinal barrier, *2*) impact on gut microbiota, *3*) human exposure data and health correlations, and *4*) dietary mitigation strategies. The strengths and limitations of evidence were considered throughout the synthesis. Although a formal risk-of-bias assessment was not performed for all studies due to their diverse nature, the methodological quality of key human observational studies was critically appraised by considering sample size, exposure assessment methods, control for confounding, and analytical validation.

## MNP Impact on Gut Barrier and Intestinal Uptake Mechanisms: A Sequential Pathway to Systemic Exposure

**FIGURE 1:**
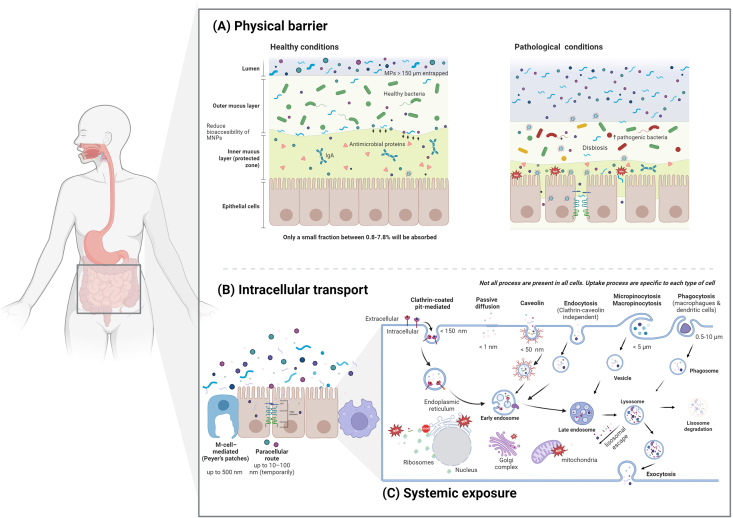
Mechanisms of intestinal interaction, transport, and cellular uptake of microplastics and nanoplastics (MNPs). (A) In the intestinal lumen, MNPs encounter a 2-layer mucus gel. The mucus layer plays a critical protective role, comprising an outer loose layer that entraps larger particles (≥150 μm) and an inner dense layer shielding epithelial cells. Under healthy conditions, mucus viscosity, antimicrobial proteins, and secretory IgA limit the bioaccessibility of MNPs, smaller particles (tens of nm–few μm) may penetrate depending on size, surface chemistry, and mucus condition. In pathological states (e.g., inflammation, dysbiosis, or increased intestinal permeability), the mucus structure and composition are disrupted, leading to reduced barrier function, enhanced translocation of MNPs, and altered host–microbiota interactions permitting greater particle–epithelial interaction. (B) Size-dependent and cell-type selective uptake pathways: Particles may traverse the barrier through *1*) M-cell transcytosis in Peyer’s patches (≤500 nm) across the epithelium; *2*) paracellular route/persoption (10–100 nm) can allow rapid entry of microparticles into the circulation under some conditions *3*) clathrin- and caveolin-mediated endocytosis internalize primarily smaller nanoplastics (tens of ∼150 nm); *4*) macropinocytosis and clathrin-/caveolin-independent endocytosis internalize submicron to low-micron particles; *5*) phagocytosis (macrophages, dendritic cells) engulf larger microplastics and mediate clearance or inflammatory signaling. *6*) Internalized MNPs traffic through endosomal compartments (early and late endosomes, lysosomes), where they may undergo degradation, exocytosis, or interact with organelles such as mitochondria and the Golgi apparatus. Particles that bypass local degradation or immune clearance can translocate into the lamina propria, enter systemic circulation via lymphatic or portal vessels, and distribute to secondary organs, posing risks for systemic toxicity. Not all uptake processes occur in every cell type; their relevance depends on particle properties (size, charge, and surface chemistry) and the physiological state of the intestinal barrier.

The GI barrier represents a critical interface between ingested MNPs through a sequential process involving physical containment, cellular transport, and potential systemic dispersal. The interaction of MNPs with biological systems begins with the interaction with the physical mucus barrier, followed by the size and cell-dependent intracellular transport and finally with the translocation leading to systemic exposure (Figure 1) [7].

### Mucus layer reduces uptake

The mucus layer constitutes the primary physical barrier against MNPs. It significantly attenuates MNPs uptake and reduces their potential detrimental effects under healthy conditions because mucus viscosity, antimicrobial peptides, and secretory IgA significantly attenuate MNP bioaccessibility of highly charged plastic particles, preventing cellular cytotoxicity and reactive oxygen species (ROS) production [8]. The barrier efficiency is size-selective; it effectively entraps larger microparticles (≥150 μm), whereas smaller nanoparticles (tens of nm) may penetrate more readily, depending on surface chemistry and mucus integrity. For example, undifferentiated Caco-2 monoculture was exposed to PS-NPs (50–500 nm), only 50 nm particles were able to translocate across the cellular barrier, and no signs of cellular toxicity were observed at any of the tested concentrations. In addition, the use of a triculture model including HT-29-MTX cells showed the mucus layer to reduce MNP bioaccessibility [9]. In contrast, PS-MNPs are also reported to alter mucus properties, forming a heterogeneous agglomeration system. They can adsorb proteins in mucus, increasing apparent particle size, and reducing surface charges. Microparticles also reduce mucus viscosity and thickness, which promotes growth of harmful bacteria, whereas NPs become mucus-entrapped, triggering secretion, disrupting microbial colonization, and inducing ROS bursts [10].

### Intracellular transport: size-dependent and cell-selective pathways

Once MNPs circumvent the mucus layer, their cellular uptake is critically determined by particle size, which dictates the transport mechanism. Meanwhile, larger plastic particles can accumulate in the GI tract, potentially impairing nutrient assimilation, compromising intestinal barrier integrity, and increasing permeability [11]. Under conditions of barrier disruption (e.g., inflammation), very small nanoparticles (10–100 nm) may traverse via the paracellular route through compromised tight junctions, a process facilitated by MNP-induced downregulation of junctional proteins like claudins and occludin. In particular, PS particles have been shown to stabilize lipid raft domains, contributing to structural disorganization and perturbing signaling pathways [12].

Smaller-sized MNPs tend to cross the epithelial barrier more easily than larger particles through transcellular translocation across epithelia, which are strongly size- and surface-dependent [13]. For example, PS-MNPs ∼50 nm in diameter are reported to enter epithelial cells via passive diffusion and energy-dependent endocytic pathways, such as clathrin-mediated endocytosis, caveolin-dependent endocytosis, and macropinocytosis. Larger particles, particularly those around 5 μm in size, are inefficiently internalized due to steric constraints and limited Brownian motion [14,15]. Particles in the 0.5–10 μm range may still be taken up by phagocytic mechanisms, whereas pinocytosis or micropinocytosis may mediate uptake of particles >1 μm [14], macropinocytosis is infrequent, but they may reach 5 μm [16]. MNPs may also bypass transcellular routes and cross the intestinal epithelium via the paracellular pathway. The size limit for molecules that can cross via the paracellular route depends on the type of epithelium and the tightness of the junctions. Under physiological conditions, tight junctions exclude particles >2 nm. Thus, paracellular MNP passage may only occur with barrier disruption (e.g., inflammation or oxidative stress), and could be exacerbated by MNP-induced intestinal barrier dysfunction, which increases paracellular permeability and enhances systemic exposure. For example, PS-NPs exposure significantly decreased the levels of tight junction proteins ZO-2, occluding, and claudin-11 by the activation of IRE1α/XBP1s/CHIP pathway [17]. In an *in vitro* study, uptake of PS particles in Caco-2 intestinal cells were apparently particle-size dependent: 0.8% at 1 μm, 3.8% at 4 μm, and 0.07% at 10 μm [7]. Also using Caco-2 cells, other studies have reported a total PS-NPs internalization ratio of 0.8%–7.8%, suggesting they may reach systemic circulation [18] at size-dependent rates [19,20].

In addition, a clear pathway for intestinal uptake of particles is mediated by M-Cell overlaying Peyer’s patches as a major portal for larger particles (≤∼500 nm) transcytosis. For example, although the translocation of 50 and 100 nm MNPs is considered to mainly depend on enterocytes (Caco-2 cells), 500 nm MNPs translocation mainly depends on M cells [21].

### Systemic exposure: translocation and distribution

Successful transcytosis across the epithelial layer allows MNPs to reach the lamina propria. MP can enter cells through various pathways and tends to predominantly accumulate within endosomes and lysosomes [15]. Lysosomal accumulation of plastics disrupts their normal function of waste digestion and leads to suppressed lysosomal activity by triggering the release of proinflammatory molecules and contributing to other adverse health effects [22]. Also, those NPs capable of endosomal escape may redistribute within the cytosol, where they can directly interact with organelles, inducing morphological alteration and structural damage [23]. For example, they can induce mitochondrial dysfunction and ATP depletion, trigger the release of ROS and cytoskeletal rearrangements [24], and interfere with endoplasmic reticulum (ER) homeostasis, leading to ER stress that ultimately contributes to apoptosis and inflammatory signaling [23,25]. Conversely, larger MPs (>1 μm) generally remain within phagosomes or extracellular spaces, where they can physically deform membranes, disrupt barrier integrity, and promote the release of proinflammatory cytokines. Overall, the intracellular fate of MNPs strongly depends on particle size, surface chemistry, and cell type; however, across systems, subcellular interactions are consistently associated with oxidative stress, metabolic dysfunction, and inflammatory activation [26].

Depending on the polymer surface chemistry, they may undergo partial biotransformation or interact more dynamically with biological systems [27], and influence the formation of a protein corona, influencing their cellular recognition, distribution patterns, and potential toxicity [28]. Excretion kinetics also vary, with smaller nanoparticles showing the potential for renal clearance, although this pathway is limited by particle aggregation and phagocytic uptake [29]. Larger MPs (>1.5 μm) are primarily retained within the GI tract and excreted via feces, although chronic exposure can lead to cumulative retention in gut-associated lymphoid tissue (GALT) and persistent local effects [15,30]. The hydrophobic nature of polymers like PE and PS promotes their sequestration in fatty tissues, leading to prolonged biological half-lives and raising concerns about chronic low-dose exposure [31]. Therefore, quantifying absorption, distribution, metabolism, and excretion (ADME) parameters for each polymer is essential for accurate risk assessment, highlighting the urgent need for standardized methodologies in both analytical detection and toxicokinetic modeling. Although comprehensive ADME data across all polymer types remain limited, synthesis of available evidence reveals distinct patterns determined by particle size, polymer composition, and surface properties.

Critically, polymer composition substantially modifies this size-dependent relationship. In a direct comparative study using Caco-2 monolayers, Stock et al. [32] demonstrated that 1–4 μm PE particles exhibited significantly higher translocation rates (2.1%–4.3%) than PS particles of equivalent size, despite similar experimental conditions. This finding challenges the common assumption that PS data can be generalized to all polymers and suggests that PE-MPs greater hydrophobicity and lower density enhance their interaction with cell membranes and uptake machinery. Also, PP showed qualitative uptake but insufficient quantitative data for rate calculation, whereas PET and PVC exhibited minimal translocation (<1%) in the micron size range [32].

### Cellular type and state

MPs uptake varies across different cell types and models, making it difficult to quantify bioavailability and potential biological effects. For example, Caco-2 cells exhibit limited PS-MP internalization, whereas uptake is much higher in THP-1 macrophages; however, both cell types have similar size affinity preferences (4 >1 >10 μm particles) [7]. In a study using differentiated and nondifferentiated Caco-2 cells, the undifferentiated cells preferentially internalized PS-MP (50 and 500 nm). The 50 nm particles translocated across the epithelial layer, reaching a maximum concentration of 0.39 μg/mL [9].

### Influence of protein corona

**FIGURE 2:**
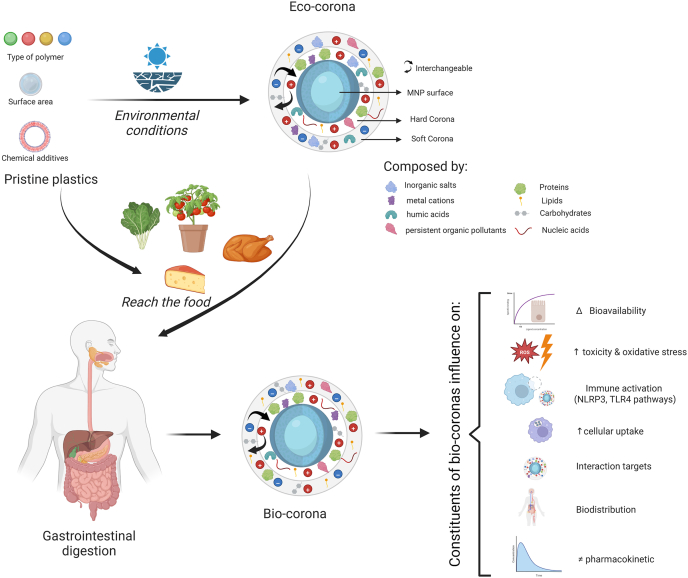
Formation and pathophysiological role of the protein corona on microplastics and nanoplastics (MNPs). MNPs initially interact with natural substances in the environment, leading to the formation of an eco-corona. On contact with biological systems, such as intestinal fluids, these particles acquire a dynamic biocorona through the adsorption of abundant biomolecules including proteins, lipids, carbohydrates, and nucleic acids, which can partially displace eco-corona constituents. The composition and structure of the corona layers (hard vs. soft) modulate key particle properties, including surface chemistry, colloidal stability, cellular recognition, and bioavailability, ultimately influencing immune interactions, cellular uptake, biodistribution, pharmacokinetics, and cytotoxicity.

On entering biological fluids, MNPs rapidly acquire a coating of adsorbed biomolecules—predominantly proteins, but also lipids and polysaccharides—forming a dynamic “protein corona” (Figure 2). This corona alters the particle’s biological identity and influences its colloidal stability, cellular recognition, and overall fate, potentially enhancing toxicity. The composition of the corona depends on factors such as polymer type, surface chemistry, particle size, and environmental conditions (e.g., ionic strengths, pH, and the presence of chemical additives) [27]. Given the hydrophobic, inert, and persistent nature of MNPs, corona formation is a key process closely linked to their migration, uptake, distribution, metabolism, clearance, and toxicity [28].

In general, particle interaction with a cell depends strongly on surface properties, including zeta potential, surface coatings [16], and conformational changes in adsorbed proteins. For example, “hard” corona consists of biomolecules strongly bound to the NP surface through electrostatic and hydrophobic interactions, which can mask surface charge and recognition sites, thereby increasing nonspecific endocytosis. In contrast, the “soft” corona is composed of loosely associated biomolecules held by reversible interactions, which can promote opsonization and facilitate phagocytic clearance [33]. The corona thus plays a critical role in determining nanoparticle transport, cellular uptake, distribution, target interactions, biotransformation, toxicity, ROS generation, immune response, and elimination [26]. For example, a “hard” corona of tightly bound proteins can mask surface charge, promoting nonspecific endocytosis, whereas a “soft” corona may facilitate opsonization and phagocytic clearance [33].

GI digestion influences protein corona formation, thereby modulating particle-cell interaction, affecting cellular internalization, and altering subsequent cellular response [34]. For example, PS-MNPs readily form stable protein coronas that enhance cellular uptake and translocation across biological barriers, which in turn exacerbate cytotoxic effects [35]. Digested PS-NPs (i.e., those with a corona) show greater cellular uptake than undigested PS-NPs across multiple cell lines [36]. Similarly, GI digestion-associated protein coronas increase the uptake of PS-MNPs by human THP-1-derived macrophages [37], with uptake rates 4.0–6.1-fold higher for uncharged MNPs <500 nm compared with larger or charged particles. Beyond influencing cellular uptake, digestion also affects the abundance of biocorona proteins involved in inflammatory processes, correlating with cytokine secretion [28]. Protein composition and the presence of additives can further impact corona formation [38,39], and should therefore be considered in MNP risk assessments. Thus, the food matrix plays a significant role in modulating NP behavior and toxicity within the GI environment [40]. For example, PS-NPs exposed to serum or plasma rapidly acquire coronas, which facilitate the MP aggregation and increase toxicity. These coronas also modulate circulation time, alter cellular uptake pathways, and reduce surface hydrophobicity, while disrupting the Keap1-Nrf2-ARE antioxidant signaling pathway and impairing the activation of antioxidant enzymes [35].

Furthermore, the corona drives inflammatory toxicity by presenting specific protein signatures that engage immune receptors (e.g., TLR4) on target cells. This engagement activates intracellular signaling cascades (e.g., NF-κB translocation, NLRP3 inflammasome assembly), leading to the transcription and release of proinflammatory cytokines (TNF-α, IL-6, and IL-1β), resulting in oxidative stress, chronic inflammation, and tissue damage [35]. In addition, there are certain polymers that may induce lysosomal damage or ROS generation, which exacerbate oxidative stress and compromise cellular redox homeostasis. In addition, when adsorbed proteins undergo conformational changes, they may lose their native function. For instance, cerebrospinal fluid (CSF) protein corona could cause the loss of active targeting specificity by shielding the targeting groups on the surface of PS-NPs and altering their cellular uptake [41]. Collectively, the protein corona acts as a central mechanistic link between MNP exposure and adverse health outcomes. By determining the “biological identity” of the particle, it directly impacts absorption kinetics (via cellular recognition and uptake) and toxicodynamic outcomes (via targeted immune receptor engagement and inflammatory pathway activation). To evaluate corona composition is therefore essential for accurate MNP risk assessment.

### MNPs as vector of cocontaminants

The role of MNPs as vectors for cocontaminants such as heavy metals, organic pollutants, and microbial pathogens merits further exploration to understand any potential synergistic or additive toxic effects. They have both inherent additives and sorbed contaminants from the environment. The large surface area, hydrophobic nature, and weathered surfaces of MNPs facilitate the strong adsorption of diverse cocontaminants, including heavy metals, persistent organic pollutants, and additives like plasticizers [42,43]. Experimental models demonstrate that this cotransport significantly amplifies biological harm. PS nanoparticles efficiently adsorb Pb^2+^ ions onto their surfaces. When tested *in vitro* and *in vivo*, this PS-Pb complex exhibits markedly greater toxicity than either component alone. The combined exposure leads to significantly higher oxidative stress, more severe intestinal barrier damage, and greater inflammatory response compared with equivalent doses of Pb or PS administered separately, suggesting the MNP delivers the metal directly to cellular interfaces [44,45]. Similarly, MNPs concentrate hydrophobic organic pollutants such as polycyclic aromatic hydrocarbons (PAHs), polychlorinated biphenyls, and plasticizers like bisphenol A (BPA). For instance, PE and PVC-MPs have a high affinity for BPA. Coexposure studies reveal that BPA adsorbed onto MPs results in more pronounced endocrine-disrupting effects, genotoxicity, and metabolic disturbance than BPA in solution. The MPs appear to act as a sustained-release platform, prolonging the bioavailability and cellular uptake of the adsorbed toxin [46,47]. This vector capacity underscores that MNP risk assessments conducted in isolation likely underestimate the true hazard. Future research must prioritize investigating the adsorption kinetics, bioaccessibility, and combined toxicodynamic pathways of these complex MNP-contaminant mixtures to fully understand their implications for human health. A frequent gap in the literature is the bridge between concentrations used in experimental studies and those encountered in real-world dietary exposure scenarios. Failure to address this gap risks overestimation of health risks and misdirection of regulatory priorities.

In this scenario, environmental monitoring provides critical benchmarks for evaluating experimental dose relevance. In aquatic systems—which ultimately supply much of the human dietary exposure through seafood—MPs concentrations typically range from 0.5 to 5.0 mg/L, with an overall increasing trend. In this context, phthalate esters such as Di(2-ethylhexyl) phthalate (DEHP), a common plasticizer and cocontaminant, have been detected in surface seawater in the Persian Gulf at mean concentrations of ∼13.7 μg/L [48].

The contribution of MPs as exposure vectors for chemicals decreases as particle size increases and ingestion rates decrease. According to predictive models, MPs can serve as vectors for chemical additive exposure. This is most significant when particles are small (1 μm) and ingested at elevated rates (≥10 mg/d). However, for the general population, their contribution to total daily chemical intake is typically minor relative to other environmental sources. The risk from MP-associated additives is greatest under high-ingestion scenarios involving particles with a high additive content (≥5% wt/wt) and becomes negligible when ingestion falls below ∼1 mg/d [49].

However, the translation of these findings to human health risk must be tempered by quantitative consideration of dose comparability. Although some recent studies demonstrate effects at environmentally relevant concentrations, others indicate that the vector effect may require exposure levels exceeding current human intake estimates by orders of magnitude. The most responsible interpretation is that cocontaminant synergy is mechanistically plausible and demonstrable in experimental systems, but its significance for human health under realistic exposure scenarios remains uncertain and requires further investigation using standardized, environmentally relevant approaches.

### Damage to colonic tissue and intestinal effects

Macroparticles and nanoparticles may affect GI function in various ways. Microparticles and nanoparticles may damage colonic barrier integrity by inducing oxidative damage and inflammation in the gut, leading to epithelial cell apoptosis [50]. They can also promote the release of proinflammatory cytokines, undermining membrane integrity and cellular functions through DNA damage [51]. This destroys the gut epithelium, reduces the mucus layer, and facilitates microbial disorders and immune cell toxicity. For example, in Caco-2 cells, in vitro administration of PVC-MPs modulates expression of genes involved in molecular binding, catalytic activity, and transcriptional regulation [52]. Also in Caco-2 cells, simulated digestion showed that at 20 μg/mL, digested PVC–MPs induce an increase in *DDIT3* and *OXR1* expression, suggesting that the cytotoxic effects of these MNPs occur by undercutting membrane integrity and producing oxidative stress [52].

*Ex vivo* analysis using irregularly-shaped PET nanoparticles (∼253 nm) revealed their rapid translocation across rat intestinal tissue, confirmed by fluorescence and Raman spectroscopy. Exposure, particularly at 10 μg/mL, altered intestinal muscle tone, indicating disrupted peristaltic function. This suggests that ingested PET nanoparticles may pose health risks through accumulation in intestinal tissue and impairment of gut motility [53].

Using a human intestine-on-a-chip model derived from healthy and Crohn’s disease donors, 25 nm polystyrene-gold core nanoparticles (AuPS25) were found to internalize via both passive and actin-/dynamin-dependent mechanisms, with minimal acute toxicity. RNA-seq revealed dysregulation of immune-related genes, notably *IFI6*, indicating potential impairment of intestinal innate immunity [53].

In addition to particle-specific factors, host physiological conditions play a critical role in modulating MNP uptake. Mice with acute colitis exhibited heightened sensitivity to PS-MPs, including exacerbated inflammation and disrupted liver metabolism, likely due to compromised intestinal barrier function. This suggests that individuals with diseases involving increased gut permeability—such as inflammatory bowel disease (IBD), celiac disease, or food allergies—may be at greater risk of enhanced MNP bioavailability and toxicity [54]. Indeed, in patients with IBD, fecal MP concentration [41.8 particles/g dry matter (DM)] was significantly higher than in healthy controls (28.0 particles/g DM). Of the fifteen identified polymers, PET (22.3%–34.0%) and polyamide (8.9%–12.4%) were the most common. This correlation suggests that dietary MPs may be associated with intestinal disease occurrence and development, or that diseases enhance susceptibility to and retention of MPs [55].

### Distribution: polymer-dependent tissue tropism

Following absorption, MNPs distribute via the circulatory system to secondary organs, with distribution patterns showing both size- and polymer-specific characteristics. PS nanoparticles distribute widely to the liver, spleen, kidney, and—in some studies—brain [30], whereas larger microparticles tend to accumulate in GALT and mesenteric lymph nodes [15,30]. The hydrophobic nature of PE and PP promotes their sequestration in adipose tissue, potentially prolonging biological half-lives [31]. This polymer-specific tissue tropism has important toxicological implications: PE accumulation in adipose creates a long-term reservoir that may slowly release particles or associated additives, whereas polystyrene’s wider tissue distribution raises concerns about direct organ toxicity.

### MNPs’ impact on human GI microbiota

The potential impact of MNPs on human gut microbiota represents an emerging area of concern, yet the underlying mechanisms remain poorly defined. On the basis of the current evidence, MNP-microbiota interactions can be categorized into 3 distinct but potentially interconnected mechanistic pathways: *1*) direct physical adhesion and biofilm formation, *2*) metabolite-mediated effects (including shifts in short-chain fatty acids (SCFAs) and plastic-derived compounds), and *3*) immune-mediated pathways that create feedback loops between mucosal inflammation and microbial dysbiosis. Understanding these mechanisms is essential for moving beyond descriptive observations toward predictive risk assessment and targeted interventions.

#### Direct physical adhesion and biofilm formation

MNPs provide a unique synthetic substrate within the intestinal lumen that facilitates direct microbial interaction. This adhesion-dependent mechanism is primarily driven by the physicochemical properties of MNPs, including surface hydrophobicity, charge, roughness, and the formation of organic coronas in the GI environment.

Evidence from in vitro fermentation models demonstrates that PET-MPs incubated with human fecal microbiota develop significant surficial organic deposits and undergo transformation into more globular surfaces, indicating active microbial colonization [56]. Similar findings have been reported for PE-MPs in animal models, where surface-adherent microbial communities form biofilm-like structures [57]. These observations suggest that MNPs function as artificial substrates that select for specific microbial taxa capable of adhering to and persisting on plastic surfaces.

The biological consequences of this adhesion mechanism are double-faced. On the one hand, biofilm formation on MNPs may protect pathogenic or opportunistic bacteria from host immune defenses and antimicrobial factors, potentially creating reservoirs of harmful microorganisms within the intestinal lumen. On the other hand, the physical occupation of MNPs within the mucus layer can disrupt normal microbial–epithelial interactions. Meng et al. [10] demonstrated that nanoparticles penetrate the protective mucus layer and become entrapped, triggering mucus secretion and disrupting normal microbial colonization patterns, whereas larger microparticles physically erode the mucus barrier and reduce the abundance of beneficial *Bacteroides* species. This size-dependent physical disruption creates ecological niches that favor opportunistic pathogens at the expense of commensal organisms. The adhesion mechanism also explains the selective enrichment of specific bacterial taxa observed across multiple studies. PE microparticles (30–140 μm) in fecal fermentation models increased abundances of *Clostridium* and *Bacteroides*—genera known for their metabolic versatility and ability to utilize diverse carbon sources—while reducing *Lactobacillus* and *Enterococcus* populations by ∼40% [46]. This selective pressure likely reflects differential adhesion capacities and metabolic adaptations to plastic-associated carbon substrates.

#### Metabolite-mediated effects: SCFA dysregulation and plastic-derived compounds

The second mechanistic pathway operates through MNP-induced alterations in microbial metabolism, leading to changes in the production of key bacterial metabolites that subsequently affect host physiology and microbial community structure. This metabolite-mediated mechanism represents an indirect but potentially profound route through which MNPs influence the gut ecosystem.

#### SCFA dysregulation

SCFAs are composed primarily of acetate, propionate, and butyrate. They are the principal metabolic products of bacterial fermentation and serve as critical mediators of host-microbe homeostasis, influencing intestinal barrier function, immune regulation, and metabolic health. Multiple studies demonstrate that MNP exposure consistently disrupts SCFA profiles, although the direction and magnitude of these changes vary with particle type, size, and exposure conditions.

In murine models, PS-MNP exposure significantly reduced cecal SCFA concentrations, particularly butyrate, with corresponding declines in butyrate-producing bacterial taxa [30,58]. This reduction in SCFAs correlates with impaired intestinal barrier function, as SCFAs (especially butyrate) are primary energy sources for colonocytes and regulate tight junction protein expression. The mechanistic link between MNP exposure and reduced SCFA production likely involves 2 interconnected processes: *1*) direct toxicity to SCFA-producing bacterial populations, and *2*) competition between plastic particles and dietary fiber substrates for bacterial fermentation.

#### Plastic-derived metabolites and carbon utilization

An emerging aspect of the metabolite-mediated mechanism involves the potential for certain gut bacteria to utilize plastic-derived carbon sources, thereby gaining a competitive advantage and altering community structure. The increased abundance of *Clostridium* and *Bacteroides* species following PE exposure has been attributed to their capacity to metabolize plastic degradation products or adsorbed organic compounds [46]. Although direct evidence of plastic biodegradation by human gut microbiota remains limited, the colonization of MNP surfaces and the formation of organic deposits [56,59] suggest that bacteria can access carbon substrates associated with weathered plastics.

Furthermore, MNPs may adsorb and concentrate dietary and host-derived metabolites, effectively sequestering them from microbial utilization or creating localized gradients that influence bacterial growth. The protein corona that forms on MNPs in intestinal fluids [34,37] includes metabolic enzymes and substrates that could be preferentially accessed by adherent bacteria, providing a competitive advantage to colonizing species. This corona-mediated metabolite concentration represents an understudied mechanism through which MNPs might locally modulate microbial metabolism.

The metabolite-mediated pathway also encompasses the potential release of plastic additives—including phthalates, bisphenols, and heavy metals—which exert direct antimicrobial effects or disrupt bacterial metabolic pathways. Although the current review focuses on the particles themselves, the leaching of additives from ingested MNPs likely contributes to the observed shifts in microbial community composition and metabolic function.

#### Immune-mediated pathways

The third mechanistic pathway involves bidirectional interactions between MNP-induced immune activation and microbial dysbiosis, creating self-reinforcing cycles that amplify GI dysfunction. This immune-mediated mechanism operates through 3 interconnected processes: *1*) direct recognition of MNPs by pattern recognition receptors on immune cells, *2*) disruption of intestinal barrier integrity that exposes immune cells to microbial products, and *3*) MNP-induced alterations in secretory IgA and antimicrobial peptide production that reshape microbial community structure.

#### Direct immune activation by MNPs

MNPs translocating across the epithelial barrier encounter mucosal immune cells, including macrophages, dendritic cells, and lymphocytes, which recognize them through various receptors. The protein corona adsorbed onto MNPs in intestinal fluids [35,37] plays a critical role in this recognition process, presenting specific protein signatures that engage immune receptors such as TLR4. This engagement activates intracellular signaling cascades such as NF-κB translocation and NLRP3 inflammasome assembly, leading to the transcription and release of proinflammatory cytokines (TNF-α, IL-6, and IL-1β) [26,35].

The resulting inflammatory milieu directly affects microbial communities. Proinflammatory cytokines alter the intestinal microenvironment by increasing oxygen tension (through increased blood flow and reduced epithelial oxygen consumption), which favors facultative anaerobes (e.g., *Enterobacteriaceae*) over obligate anaerobes (e.g., *Firmicutes* and *Bacteroidetes*). This cytokine-mediated shift explains the consistent observation across multiple studies of increased *Enterobacteriaceae* and *Desulfovibrionaceae* following MNP exposure [60,61].

#### Barrier disruption and microbial translocation

MNP-induced damage to intestinal barrier integrity, as previously discussed, creates a second immune-mediated feedback loop. Disruption of tight junction proteins (ZO-2, occludin, and claudin-11) via pathways such as IRE1α/XBP1s/CHIP activation [17] increases paracellular permeability, allowing luminal bacteria and their products (e.g., lipopolysaccharide, flagellin) to translocate into the lamina propria. This microbial translocation amplifies local inflammation, which further compromises barrier function and alters microbial community structure.

The high sensitivity to MNP toxicity observed in individuals with IBD [55] and in murine models of colitis [54] exemplifies this bidirectional amplification loop. Pre-existing inflammation compromises barrier function, increasing MNP translocation; MNPs then exacerbate inflammation through direct immune activation and additional barrier disruption. This vicious cycle likely explains the significantly higher fecal MP concentrations (41.8 compared with 28.0 particles/g DM) observed in patients with IBD compared with healthy controls [55].

#### Disruption of secretory IgA and antimicrobial peptides

A third immune-mediated pathway involves MNP-induced alterations in mucosal humoral immunity. Secretory IgA and antimicrobial peptides (e.g., defensins) represent the front line of immune defense, shaping microbial community composition by limiting bacterial adhesion and epithelial association. Zhang et al. [30] demonstrated that PS–MNP exposure in mice significantly reduced intestinal IgA levels and impaired T cell differentiation, with NPs exhibiting more severe effects than MPs.

The reduction in IgA creates ecological opportunities for bacteria that would otherwise be excluded from the epithelial niche, favoring adherent and potentially pathogenic species. This mechanism explains, in part, the overrepresentation of opportunistic bacteria—including *Enterobacteriaceae*, *Desulfovibrio* spp., and *Clostridium* group I—observed following MNP exposure [60,61]. The concurrent depletion of beneficial taxa such as *Lactobacillus* and *Bifidobacterium*, which normally stimulate IgA production and maintain immune homeostasis, further amplifies this dysregulation.

#### Integration of mechanistic pathways

These 3 pathways—direct adhesion, metabolite-mediated effects, and immune-mediated pathways form an integrated network. Adherent bacteria on MNP surfaces are exposed to locally concentrated nutrients and metabolites, while simultaneously encountering a microenvironment shaped by MNP-induced immune activation. The protein corona that forms on MNPs during intestinal transit [28,37] serves as a molecular interface that connects all 3 pathways: corona composition influences bacterial adhesion, determines metabolite adsorption, and dictates immune cell recognition.

### Human evidence and mechanistic understanding

Translation of these mechanistic insights to human health remains limited by the scarcity of well-controlled human studies. However, emerging evidence supports the relevance of these pathways in human populations. Ke et al. [62] demonstrated that preschool children with higher fecal MP concentrations exhibited lower abundances of *Lactobacillales*, *Rikenellaceae*, *Alistipes*, and *Streptococcus*—genera associated with SCFA production and anti-inflammatory effects. The negative correlations between PVC and A*listipes*, and between PE and *Parabacteroides*, suggest polymer-specific effects that likely operate through differential adhesion capacities or corona compositions.

Overall, direct evidence linking fecal MP content to gut microbiota composition remains sparse. More robust, well-controlled human studies are needed to identify potential mechanisms of interaction and to map the extent of MNP involvement in gut microbial modulation.

The detection of multiple polymer types in human feces across diverse populations (Table 1 [39,42,58,63,64,67,68]) confirms that ingested MNPs reach the colonic microbiota, making physical interaction inevitable.

**TABLE 1:** Recent studies reporting microplastics in human stools

Reference	Detection and sample numbers	Size/concentration	Polymer type	Shape	Ratio of incidence and conclusions
Schwabl et al., 2019 [63]	FT-IR *n =* 8	50–500 μm $\bar{X}$ = 20 MPs/10 g	9 MP types detected: PP = 62.8% PET = 17%.	Fragments and films; rare spheres or fibers	Ubiquitous. Inadvertently ingested from various sources.
Zhang et al., 2021b [64]	FTIR *n =* 26	20–800 μm 1–36 particles/g 0.01–14.6 mg/individual	8 MP types detected: PP = 95.8% PET PS PE PVC PC PA PU	n.d.	MPs detected in 23/24 (95.8%) participants’ feces.
Refosco et al. 2025 [65]	μ-FTIR *n =* 18 (seafood consumers vs. nonconsumers)	12.5–4065 μm	3 MP types detected: PP (72%) PE (44%) PS (16%)	n.d.	Detected in 17/18 samples Smaller microplastics (10–50 μm) are the most abundant size class.
Hartmann et al. 2024 [66]	FTIR *n* = 15	n.d. 3.5 particles/g stool (in 50–500 μm fraction) 0.26 and 49 particles/g.	PE and PET most frequently detected (>60%). Detection frequency 8% for PMMA and 68% for PE.	Fragments and fibers most frequent	≤9 different plastic types detected in stool samples.
Ho et al. 2022 [67]	Raman spectroscopy *n = 8*	40.2–4812.9 μm (88% <300 μm) 129 MPs total	PS PP PE	Primarily fragments, some fibers (PET was 2/3 of the total fiber content)	Most abundant particle was PS (50 particles/μg), followed by PP and PE.
Luqman et al., 2021 [68]	Raman spectroscopy *n =* 11	$\bar{X}$ = 9.195 3.33–13.99 μg/g feces	HDPE 9.195 μg/g (the most prevalent) PS LDPE LLDPE PP PET	n.d.	MPs identified in >50% samples.
Wibowo et al., 2021 [39]	Raman spectroscopy *n =* 11	$\bar{X}$ = 10.19 μg/g feces	PP was the most abundant and prevalent type of microplastic observed, and it was found in 4 of the positive samples	n.d.	MPs in 7 of 11 samples MP contamination in the rural Indonesian population and in their daily consumables demonstrates the far-reaching extent of MP pollution beyond urban areas.

Abbreviations: FT-IR, Fourier-transform infrared spectroscopy; HDPE, high-density polyethylene; LDPE, low-density polyethylene; LLDPE, linear low-density polyethylene; MPs, microplastics; NPs, nanoplastics; PA, polyamide; PC, polycarbonate; PE, polyethylene; PET, polyethylene terephthalate; PMMA, polymethyl methacrylate; PP, polypropylene; PS, polystyrene; PVC, polyvinyl chloride; μ-FTIR, micro-Fourier-transform infrared spectroscopy; n.d., not determined; $\bar{X}$, mean value.

All the studies showed consistent patterns in human fecal MNP profiles, although the studies showed a significant methodological heterogeneity. The reported concentrations vary widely, ranging from ∼0.26 to 49 particles per gram of stool (dry or wet weight), with many studies reporting means between 10 and 20 particles/g [53,63,69]. This variability reflects differences in exposure sources, geography, and individual diet, as well as analytical techniques. Regarding polymer composition, PP, PET, and PE are consistently identified as the most prevalent types across multiple cohorts, often collectively accounting for over 70%–80% of detected particles [63,64,67]. In terms of particle size, although early studies focused on the >50 μm fraction due to methodological constraints, more recent work employing advanced spectroscopy indicates a high abundance of smaller particles (<300 μm), suggesting earlier data may underestimate total burden [70].

However, the interpretation of these data must be tempered by acknowledging several key study limitations. First, the sample size in these human excreta studies is generally small (typically *n* < 30), limiting statistical power and the generalizability of findings to broader populations. Second, there is substantial detection and reporting bias. Studies employ different analytical techniques (e.g., μ-FTIR compared with Raman spectroscopy) with varying size detection limits, sensitivities, and polymer identification libraries, making direct cross-study comparisons challenging [53,70]. For example, methods optimized for larger microparticles may miss the NPs fraction, which is potentially more bioavailable. In addition, reporting units are inconsistent, with some studies reporting particle counts and others mass concentrations (μg/g), which are not directly convertible without assumptions about particle size and density. Furthermore, the risk of sample contamination during collection, processing, and analysis is a persistent concern that can lead to overestimation, underscoring the need for stringent procedural blanks and standardized protocols [66]. Lastly, the available human studies are predominantly cross-sectional and observational, capturing only a snapshot in time; they cannot establish causality or delineate the kinetics of MNP ingestion, retention, and excretion. These limitations collectively highlight that current fecal MNP data, while providing crucial proof of exposure, represent a preliminary and likely incomplete picture, emphasizing the urgent need for larger, longitudinal studies using harmonized, sensitive, and validated methodologies.

Although increasing, current data on the effects of MNPs on the human GI microbiota remain largely descriptive and fragmented. Future research must prioritize human studies that integrate MNP exposure assessment with metagenomic profiling, metabolomics (particularly SCFA quantification), and mucosal immune markers. Such integrated approaches will enable testing of the mechanistic framework proposed here and identification of intervention targets. The development of standardized analytical methods for MNP detection in biological samples is a prerequisite for these studies, as current methodological heterogeneity limits cross-study comparability and mechanistic interpretation.

## Diet and MNPs

Humans are at the top of the food chain and thus exposed to plastic fragments through the consumption of contaminated dietary products [71,72]. Water consumption is a key determinant of daily MNP intake. Tap water typically contains less plastic (0–660 MP/d) than bottled water (0.0091–1,531,524 MP/d). In the latter, contamination originates mainly from the packaging and bottling processes [73]. Although these values vary geographically, they underscore the advantage of drinking tap water (if it is safe) and minimizing reliance on bottled beverages.

As mentioned, food processing and packaging impact MNPs exposure. In general, concentrations (MPs/g food) differ between levels of processing, with highly processed products containing significantly more MPs than minimally processed ones [74]. Prolonged storage, heating, and use of disposable plastic containers also increase MNP migration into food [66], and may facilitate leaching of other contaminants such as phthalates. The extent of this transfer is influenced both by packaging material type and food physicochemical properties [75]. To reduce exposure, food choices should focus on avoiding ultraprocessed and highly packaged foods, and preferring unpackaged alternatives; dietary patterns emphasizing fresh, plant-based foods are generally healthier, anyway [76,77].

Humans occupy high trophic positions and are thus exposed to MNPs not only through direct contamination of food and water but also through the consumption of organisms that have accumulated plastics from their own prey [67,71].

MNPs enter food webs by direct environmental uptake by organisms at low trophic levels, and by the subsequent predator-prey transfer. At the base of aquatic food chains, filter-feeding organisms (zooplankton, bivalve mollusks) and deposit-feeders inadvertently ingest suspended or settled MNPs [78]. These organisms cannot distinguish plastic particles from their natural food sources due to similar size ranges and once ingested, particles may be retained in digestive tracts, translocated to tissues, or excreted, with retention efficiency depending on particle characteristics and organism physiology [79].

The transfer to higher trophic levels occurs when contaminated prey are consumed by predators [80]. The efficiency and extent of trophic transfer depend on multiple interacting factors, with particle size being paramount. Nanoparticles (<1 μm) exhibit greater potential for translocation across biological barriers and accumulation in internal tissues, whereas larger MPs (>10 μm) tend to remain within the GI tract.

This process can lead to bioaccumulation (increasing contaminant concentration within an organism over time) within organisms and potential biomagnification (increasing concentration across trophic levels) across food webs [71,72]. The efficiency and extent of trophic transfer depend on multiple interacting factors, with particle size being the most important. For instance, nanoparticles (<1 μm) exhibit greater potential for translocation across biological barriers and accumulation in internal tissues, whereas larger MPs (>10 μm) tend to remain within the GI tract [81]. In addition, particle shape influences transfer dynamics. Fibers may exhibit different retention and translocation patterns compared with spheres or fragments due to their ability to entangle in GI structures. Polymer type also determines density, which affects both environmental distribution and residence time in organisms [78]. Surface properties and weathering status modulate trophic transfer through their effects on particle aggregation, protein corona formation, and cellular interactions. Weathered particles with altered surface chemistry may exhibit different bioaccumulation potentials compared with pristine laboratory-grade particles [81].

On the other side, organism-specific factors profoundly influence MNP accumulation and transfer. A recent meta-analysis examining contaminant accumulation across marine species found strong correlations between MP concentrations and trophic level, indicating systematic transfer through food webs [82]. In addition, lifespan significantly predicts MNP accumulation, with longer-lived organisms showing greater body burdens due to prolonged exposure and limited elimination. Also, other characteristics like feeding mode and digestive physiology affect particle degradation and elimination.

**FIGURE 3:**
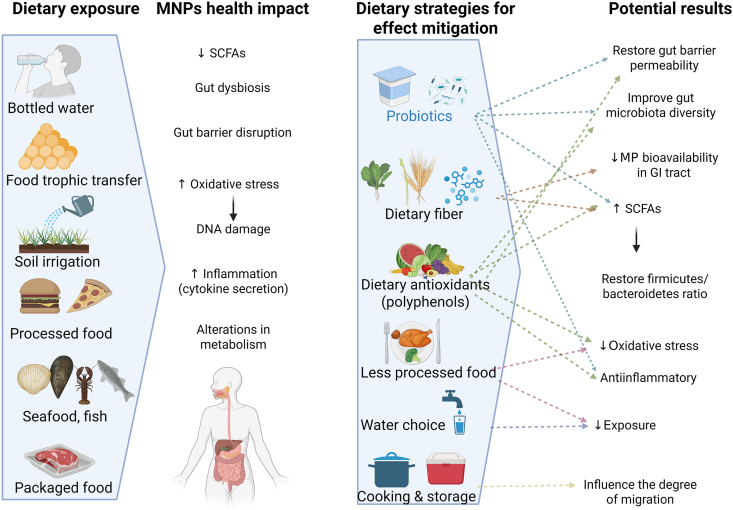
Dietary strategies focused on the mitigation of the deleterious effects of MNPs. GI, gastrointestinal; MNP, microplastics and nanoplastics; MP, microplastics; SCFA, short-chain fatty acids.

Consequently, dietary habits, therefore, play a pivotal role in modulating both the extent of MNP intake and the biological consequences of exposure (Figure 3).

At the same time, certain dietary compounds may reduce the potential toxicity of MNPs through different interconnected physiological mechanisms that converge on enhancing gut barrier integrity and dampening inflammatory signaling. In vitro and animal studies suggest that dietary fiber may lower MP bioavailability in the GI tract by adsorbing and entrapping particles, as well as by reducing intestinal transit time, thus potentially facilitating fecal elimination. A recent study in rats demonstrated that chitosan, a nondigestible fiber, significantly increased fecal excretion of ingested PE-MPs over 1 wk [83]. However, human data confirming this effect are currently lacking.

Paradoxically, commercial fiber supplements may be contaminated with MPs, resulting in a possible ingestion rate of around 5.9 ± 2.9 particles/d [84]. Fiber also exerts protective effects by supporting gut microbiota homeostasis through its prebiotic-like effects. Fiber supplementation restores SCFAs production and reverses MNP-induced dysbiosis, suggesting that maintaining optimal *Firmicutes*/*Bacteroidetes* ratios altered by MP exposure [85], mitigating MP-induced dysbiosis and their metabolic consequences [56].

In addition, experimental studies in vitro and in animal models indicate that dietary antioxidants such as (poly)phenols may ameliorate potential deleterious effects induced by MP-induced oxidative stress and inflammation. For example, supplementation with cyanidin-3-O-glucoside exhibits antioxidant, anti-inflammatory, and microbiota-modulating effects, reducing the effects produced by PS particle accumulation in tissues [86]. Similarly, the flavonoid naringin has demonstrated therapeutic potential in protecting the endocrine system from PE-MPs toxicity. Specifically, oral coadministration of naringin (100 mg/kg) in mice exposed to MPs (1.5 mg/kg) significantly reversed hormone imbalances and restored Kallikrein-3 levels. This protective effect was driven by the upregulation of antioxidant defenses (GSH, SOD, and CAT) and the suppression of MDA, effectively neutralizing ROS production and subsequent systemic inflammation [87]. Also, (Poly)phenols also exert a prebiotic-like effect [88], enhancing the production of different SCFAs, primarily acetate, propionate, and especially butyrate, which display protective barrier effects by enhancing the barrier function, exert immunomodulation, create a mildly acidic luminal environment that favors beneficial bacteria and inhibits pathobionts as well as attenuate MNP-induced dysbiosis and toxicity.

Also, other bioactive compounds like carotenoids may impact the mitigation of oxidative stress. A recent study in European seabass juveniles demonstrated that dietary microencapsulated astaxanthin (a carotenoid with potent antioxidant properties) mitigated MP-induced oxidative stress and promoted MP coagulation in the gut, limiting tissue accumulation [89].

So, although human data are limited, and controlled human studies are lacking, experimental studies in vitro suggest that dietary antioxidants may mitigate MP toxicity through the activation of the nuclear factor erythroid 2-related factor 2 pathway and stress-resilience proteins at minimum doses, thereby preventing or blocking MP and NP-induced damage [90].

Emerging evidence from preclinical models suggests that targeted dietary strategies do not merely reduce exposure but actively engage the host-microbiota axis to build physiological resilience, offsetting the oxidative, inflammatory, and dysbiotic consequences of unavoidable MNP intake. Low-dose MP exposure and/or a high-fat diet significantly increase gut permeability, oxidative stress, proinflammatory response, and apoptosis, while concurrently contributing to gut dysbiosis (e.g., reduced levels of *Akkermansia*) and development of chronic diseases [31]. Although no intestinal probiotic bacteria capable of degrading plastic have yet been identified [91], they can be considered as both a preventive measure and a treatment of the negative effects of MNPs. They may provide multiple benefits, with certain strains capable of biosorbing or degrading cocontaminants such as heavy metals and bisphenol A, whereas promoting MNP aggregation to limit bioavailability. These findings derive primarily from in vitro and animal studies and may not reflect real-world human scenarios. They suggest their potential biological potential [91].

Preliminary evidence from in vitro screens and a single mouse study suggests that strain-specific probiotics may represent a promising strategy for mitigating MP-associated health risks. For example, *Lacticaseibacillus paracasei* DT66 and *Lactiplantibacillus plantarum* DT88 demonstrated high adsorption capacity for PS particles in vitro, and in mice, DT88 administration reduced intestinal MP burden and alleviated MP-induced inflammation [38]. These findings warrant further investigation but cannot yet support recommendations for human use. Overall, the dietary strategies discussed above are supported by a growing body of preclinical evidence demonstrating plausible mechanisms for mitigating MNP toxicity. However, the translational gap between these models and human applications remains substantial.

## Future MNPs Research in Human Health

Evidence of MNP effects in human tissues and organs is limited due to the use of preclinical models, including in vitro and animal studies. To transition from hazard identification to credible risk assessment, future research must adopt structured, interdisciplinary frameworks. For instance, a primary obstacle is the lack of standardized, sensitive analytical methods for MNPs in complex biological matrices; for that, it is necessary to coordinate labs worldwide to develop, validate, and harmonize techniques capable of detecting and quantifying MNPs in human tissues, fluids, and excreta. Advances in rapid, high-throughput screening technologies will also be essential for assessing real-time distribution and accumulation patterns in biological systems.

The toxicokinetic principle that “smaller is more bioavailable” requires precise quantification. Research must establish size-dependent absorption rates (ADME) for common polymers at environmentally relevant doses. In addition, emerging toxicokinetic studies underscore that the ADME of MNPs is profoundly influenced by their physicochemical properties, particularly particle size and polymer composition.

The mechanistic basis for these polymer-specific differences likely involves multiple factors: surface hydrophobicity influences membrane partitioning and protein corona composition [27,28]; particle density affects sedimentation kinetics and cell-particle contact frequency; and surface charge modulates electrostatic interactions with negatively charged cell membranes. These observations underscore the inadequacy of PS-only models and the urgent need for systematic comparative studies across common polymers using standardized methodologies.

Current evidence suggests that mammals lack enzymatic systems capable of significant plastic polymer degradation. The carbon–carbon backbones of PE, PP, PS, and PET are resistant to hydrolysis by mammalian enzymes, and no studies have demonstrated substantial polymer chain cleavage in mammalian tissues. Some in vitro evidence suggests possible surface oxidation of PS via cytochrome P450 enzymes in bacteria, but this represents minor chemical modification rather than true metabolism that would reduce particle size or facilitate elimination [92].

New experimental designs should move beyond pristine, spherical PS particles. Research frameworks must test environmentally relevant mixtures, including weathered particles of common polymers (PE, PP, and PET), a range of sizes (with emphasis on the nanoscale), and in combination with adsorbed cocontaminants (heavy metals, PAHs) at concentrations relevant to human exposure.

Prospective studies have examined the potential role of MNPs in different disease conditions, but these are preliminary and often lack standardized methodologies, precluding robust conclusions. No epidemiological studies exist that correlate MNP exposure with different diseases and assess their potential long-term impacts. Longitudinal cohort studies and the use of different biobanks will help to evaluate the potential health risks associated with chronic low-dose exposure, and correlated with subsequent disease onset, while controlling for key confounders like diet and lifestyle, particularly in vulnerable populations.

In addition, cohort studies should systematically collect data beyond MNP concentration. A dedicated framework integrating MP burden with host multiomics (metagenomics of gut microbiota, metabolomics of serum/feces, inflammatory proteomics) and deep phenotyping (clinical diagnostics, gut permeability tests) is necessary to disentangle MNP-specific effects from general environmental correlations and to elucidate mechanistic pathways in humans.

The potential involvement of MNPs in disease pathogenesis, especially oxidative stress and inflammatory conditions, is an important focus. Although existing human data are limited, preclinical evidence highlights the need for interdisciplinary research aimed at characterizing MNP biodistribution, accumulation, toxicity mechanisms, and health outcomes.

Mechanistic research will elucidate how MNPs modulate microbiota and determine if shifts in microbiota translate into adverse health outcomes or have broader ecological impacts. Human in vivo studies using environmentally relevant exposure levels and robust metagenomic and metabolomic approaches will be essential to understanding MNP-microbiota interactions and their public health implications.

The field should adopt a standardized framework utilizing advanced in vitro models (e.g., gut-on-a-chip with integrated mucus, epithelium, immune cells, and microbiome) as a prerequisite for animal studies. These models can efficiently screen for mechanisms of barrier disruption, immune activation (e.g., NLRP3, TLR4 pathways), and microbiota dysbiosis under controlled conditions. Experimental designs should move beyond pristine, spherical, and move from PS particles. Research frameworks must test environmentally relevant mixtures, including weathered particles of common polymers (PE, PP, and PET), a range of sizes (with emphasis on the nanoscale), and in combination with adsorbed cocontaminants (heavy metals, PAHs) at concentrations relevant to human exposure.

Food and beverage packaging is a major source of MNP contamination, underscoring the need for advanced filtration technologies, safer packaging materials, and stricter quality control. Research highlights the value of standardized detection methods, risk assessment of plastic-associated chemicals and microbes, and consumer education about sustainable packaging. For that, it would be necessary to design and conduct randomized controlled trials or carefully controlled dietary intervention studies to test the efficacy of specific strategies (e.g., a diet low in ultraprocessed foods, high in specific fibers, or supplemented with defined probiotic strains) in reducing fecal MNP excretion or biomarkers of MNP-associated inflammation in high-risk groups (e.g., patients with IBD).

Public health strategies should consider dietary and lifestyle interventions to reduce GI exposure to plastics. These can include promotion of dietary patterns that minimize consumption of highly processed, packaged foods, and inclusion of protective components that may mitigate MNP exposure-associated oxidative and inflammatory responses. Also, they should promote the use of sustainable packaging and promote the use of different material innovation systems. These recommendations must be supported by upstream policy and industry action to reduce environmental contamination and foodborne exposure. International bodies such as FAO, WHO, and the European Union, among others, should develop public health strategies to empower consumers, as well as to support the policy-driven systemic change.

In conclusions, dietary intake is a major route of microparticle and nanoparticle exposure in humans. These particles contaminate a vast array of foodstuffs and beverages, with migration from plastic packaging constituting a significant additional source. In vitro and animal studies have demonstrated that MNPs can compromise GI health by inducing oxidative stress, disrupting epithelial barrier integrity, altering digestive enzyme activity, and causing gut microbiota dysbiosis. These outcomes are mainly determined by factors such as particle size and type, dosage, and surface properties. Alterations may be more pronounced in high-risk populations such as individuals with IBD. Disruption of the colonic mucus barrier may further facilitate translocation of MNPs into the bloodstream, contributing to systemic toxicity.

The current data may suggest negative health effects from MNPs, but there is a stark disconnect between demonstrated exposure and proven pathogenicity in humans. Provocative correlations have linked MNP exposure to conditions such as IBD and cardiovascular disease, but causality has not been established. Nonstandardized detection methodologies, sample contamination risk, and reliance on high-dose, nonphysiological experimental models prevent accurate conclusions. Future research priorities are clear. Standardized analytical techniques must be developed, toxicokinetic studies at environmentally relevant doses are lacking, and rigorous epidemiological cohorts. Barring extensive future research, any declared potential health risk of MNPs in humans should be taken with caution. In the meantime, generally reducing plastic use, particularly in food systems, would be a wise strategy, as is a deeper investigation into dietary modulation of MNP bioavailability and toxicity.

## Funding

This research was funded by MCIN/AEI/10.13039/501100011033 through the European and ERDF A way of making Europe (PID2023-150666OA-I00), by the Ramon Areces foundation (CIVP21S13273), and by Comunidad de Madrid EXOHEP2-CM (S2022/ BMD-7409). M-CLdlH is a recipient of a Ramon y Cajal Grant RYC2022-037626-I. VM-S is supported by a predoctoral fellowship from Comunidad de Madrid (PIPF-2023/SAL-GL-30783). CIBEROBN (CB22/03/ 00068) is an initiative of the Instituto de Salud Carlos III, Spain.

## Author contributions

The authors’ responsibilities were as follows – AD, M-CLdlH: conceptualized the paper; M-CLdlH: wrote the first draft of the manuscript, and all authors: collected and analyzed data, read and approved the final version of manuscript.

## Declaration of generative AI and AI-assisted technologies in the writing process

No generative AI or AI-assisted technologies were used during the drafting, writing or editing of this manuscript.

## Conflict of interest

The authors report no conflicts of interest.
